# Verbesserung der Atemmechanik durch Plattenosteosynthese der Rippen nach Herzdruckmassage: Fallbericht und Literaturübersicht

**DOI:** 10.1007/s00113-020-00950-z

**Published:** 2021-01-12

**Authors:** Karl Friedrich Abshagen, Josef Stolberg-Stolberg, Jan Philipp Loyen, Oliver Riesenbeck, Jens Everding, Hendrik Freise, Michael J. Raschke

**Affiliations:** 1 Klinik für Unfall‑, Hand- und Wiederherstellungschirurgie, Universitätsklinikum Münster, Waldeyerstraße 1, 48149 Münster, Deutschland; 2 Klinik für Anästhesiologie, operative Intensivmedizin und Schmerztherapie, Universitätsklinikum Münster, Albert-Schweitzer-Campus 1, Gebäude A1, 48149 Münster, Deutschland

**Keywords:** Thoraxstabilisierung, Reanimation, Inverse Atmung, ORIF-Rippenserienfraktur, Atemmechanik, Flail chest stabilization, Resuscitation, Inverted breathing, Multiple rib fractures, ORIF, Breathing mechanics

## Abstract

Es wird der Fall eines 69-jährigen Patienten vorgestellt, welcher nach wiederholter Herzdruckmassage (CPR) multiple Rippenfrakturen und eine Sternumfraktur mit inverser Atmung zeigte. Aufgrund der insuffizienten Atemmechanik wurde am 10. Tag nach Reanimation die Indikation zur Plattenosteosynthese der Rippen gestellt. Der Patient konnte am 4. postoperativen Tag extubiert werden. Die Literatur zeigt 5 weitere Fälle. Während es über die Verletzungsmuster nach CPR zahlreiche internationale Arbeiten gibt, ist zur Behandlung des instabilen Thorax nach CPR keine Evidenz gegeben. Jedoch konnte die Osteosynthese von Rippenfrakturen bei traumatischer Genese Vorteile wie verringerte Intensivbehandlungszeit, geringere beatmungsassoziierte Pneumonien, weniger atemabhängige Schmerzen und geringere Kosten für das Gesundheitssystem zeigen. Bei der Behandlung von reanimierten Patienten sollte im interdisziplinären Konsens auch die Osteosynthese von Rippenfrakturen berücksichtigt werden.

**Zusatzmaterial online:**

Die Online-Version dieses Beitrags (10.1007/s00113-020-00950-z) enthält ein Video, das die inverse Atmung zeigt. Beitrag und Zusatzmaterial stehen Ihnen auf www.springermedizin.de zur Verfügung. Bitte geben Sie dort den Beitragstitel in die Suche ein, das Zusatzmaterial finden Sie beim Beitrag unter „Ergänzende Inhalte“.

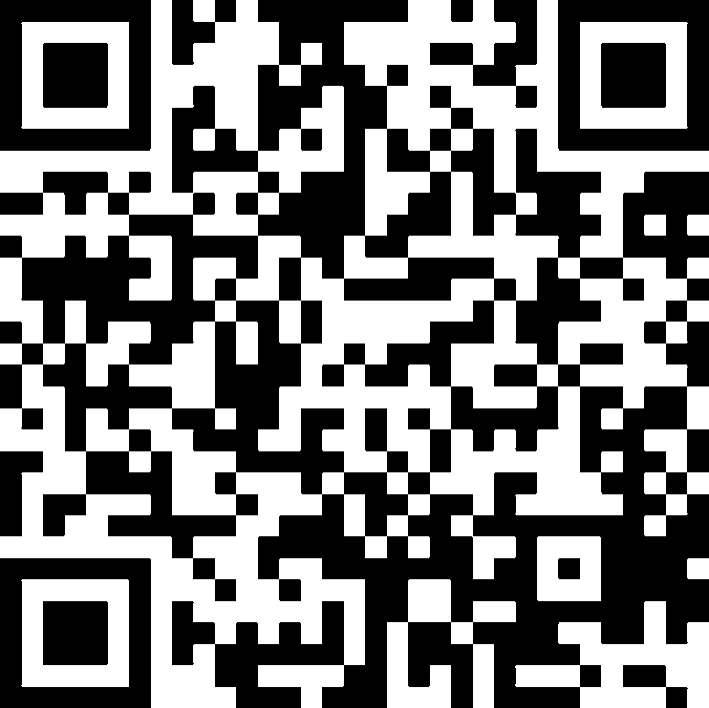

## Abstract (en)

This article reports the case of a 69-year-old patient with multiple rib fractures and sternal fracture after repetitive cardiopulmonary resuscitation (CPR). Because of secondary respiratory failure due to an unstable thorax, rib fixation was performed 10 days after CPR. Subsequently, ventilation improved resulting in successful extubation 4 days after rib plating. A review of the literature revealed only five documented cases of rib osteosynthesis after CPR. Although flail chest occurs in up to 15% of patients after CPR, there is little evidence of the effect of rib fixation. The benefit of this procedure after chest trauma is reduced pain, shortened intensive care unit stay, lower rates of ventilation-associated pneumonia and lower costs for the healthcare system. Further clinical research is needed and interdisciplinary treatment should be kept in mind when dealing with patients resuscitated with prolonged mechanical ventilation.

## Anamnese

Ein 69-jähriger Patient erlitt im Rahmen eines Nicht-ST-Hebungs-Infarkts einen Herzstillstand. Nach 10-minütiger Laienreanimation wurde durch den Notarzt nach Defibrillation eine Spontanzirkulation wiederhergestellt. Eine am selben Tage durchgeführte Koronarangiographie ergab eine Eingefäßerkrankung. Nach Ausschluss behandlungsbedürftiger Stenosen wurde nicht weiter interveniert. Am 2. Tag nach Aufnahme wurde der Patient erneut mehrfach reanimationspflichtig und daraufhin intubiert. In einer erneuten Koronarangiographie erfolgte die Implantation von 2 „drug-eluting stents“. Bei inverser Atmung erfolgte am 3. Tag die Extubation mit zunächst suffizienter Spontanatmung. Am 6. Tag wurden bei einem mechanischen Ileus eine Hemikolektomie rechts mit End-zu-End-Anastomose sowie eine Cholezystektomie durchgeführt. Im Anschluss an den Eingriff konnte der Patient zunächst noch im OP extubiert werden. Im Laufe des folgenden 7. Tages entwickelte der Patient im Rahmen des septischen Mehrorganversagens zunächst eine progrediente Oxygenierungsstörung und erschöpfte sich schließlich mit Tachypnoe, Hyperkapnie und konsekutiver Somnolenz, sodass die erneute Intubation erfolgte. Eine „Second-look“-Laparotomie am 8. Tag zeigte eine persistente akute Peritonitis.

## Klinischer Befund

**Figure 1:**
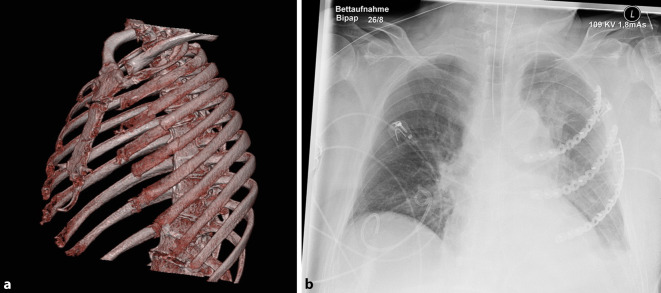

Durch die mehrfache Reanimation zog sich der Patient eine Sternumfraktur, Mehrfragmentfrakturen der 3. bis 5. Rippe sowie eine einfache Fraktur der 2., 6. und 7. Rippe links zu. Außerdem zeigten sich nichtdislozierte Rippenserienfrakturen 2–5 rechts (Abb. 1). In der Projektionsradiographie und in angeschnittenen Lungenanteilen im Abdomen-CT (Computertomograph) zeigte sich eine links basal führende Pneumonie mit erheblicher Sekretretention. Es bestand ein moderates ARDS (Acute Respiratory Distress Syndrome) mit Horowitz-Indizes zwischen 100 und 150 mmHg unter druckkontrollierter Beatmung mit Möglichkeit zu druckunterstützter Spontanatmung. Die Decarboxylierung war unter lungenprotektiven Beatmungsparametern ungestört.

Zum Indikationszeitpunkt (10 Tage nach erstmaliger Reanimation) blieben Spontanatmungsversuche allerdings frustran. Es zeigte sich klinisch ein zentraler instabiler Thorax mit inverser Atmung (Zusatzmaterial online: Video 1) und Sauerstoffpartialdrücken von 60 mm Hg bei einer F_I_O_2_ von 55 %. Weiterhin zeigten sich steigende Infektparameter und in der radiologischen Bildgebung (Abb. 1) eine Pneumonie mit linksbetonten Dystelektasen. Daher wurde am 10. Tag die Indikation zur Plattenosteosynthese der Rippen gestellt.

## Diagnose

Inverse Atmung bei

- nichtdislozierter Rippenserienfraktur 2–5 rechts,
- Mehrfragmentfrakturen der 3.–5. Rippe links,
- dislozierten Einfachfrakturen der 2., 6. und 7. Rippe links,
- Querfraktur des Sternumkorpus im 3. ICR (AO 16.3.2 A)

bei Z. n. kardiopulmonaler Reanimation.

## Therapie und Verlauf

Am 11. Hospitalisationstag nach Herzstillstand erfolgte die osteosynthetische Versorgung der Rippenfrakturen mittels offener Reposition und Fixierung (MatrixRib, Fa. Synthes, Oberdorf, Schweiz). Intraoperativ zeigte sich bereits nach Stabilisierung der Rippen 3–5 links ein Ausbleiben der inversen Atmung in einem ersten Spontanatemversuch. Im konventionellen Thoraxröntgen konnte postoperativ ein Pneumothorax ausgeschlossen werden. Am 1. postoperativen Tag persistierte unter dem klinischen Bild einer rückläufigen Peritonitis und einer *E.-coli*-Pneumonie noch eine deutliche Oxygenierungsstörung. Ab dem 2. Tag wurde unter erheblich gebesserter Atemmechanik und effektivem Hustenstoß mit dem Weaning begonnen, und ab dem 3. Tag konnte eine vollständig spontane Atmung mit einem PEEP („positive end-expiratory pressure“) von 7 mbar erreicht werden. Nach Resolution eines hyperaktiven Delirs konnte am Tag 4 die Extubation erreicht werden. Es zeigte sich eine suffiziente Eigenatmung unter Raumluft mit reduzierten Schmerzen.

## Diskussion

Zunehmende Evidenz belegt die Effektivität der Behandlung des instabilen Thorax durch Plattenosteosynthesen. Aktuelle Richtlinien empfehlen die Erwägung einer Rippenstabilisierung bei instabilem Thorax, Rippenserienfrakturen > 3 Rippen, grober Dislokation und symptomatischer Pseudarthrose [1]. Der instabile Thorax ist definiert als Verletzung mit mehr als 2 Frakturstücken/Rippe in mindestens 3 aufeinander folgenden Rippen [2]. Mittlerweile konnte in 3 kontrollierten randomisierten Studien gezeigt werden, dass die Rippenverplattung bei Patienten mit instabilem Thorax folgende Vorteile zeigt: kürzere Beatmungsdauer, kürzere Intensivtherapie, niedrigere Pneumonieraten, verbesserte Lungenfunktion, Abnahme der posttraumatischen Schmerzen sowie reduzierte Kosten für das Gesundheitssystem [1, 3]. Ursächlich für ein Thoraxtrauma wird meist das Hochrasanztrauma beschrieben. In 6 % dieser Fälle resultiert aus dem Unfall ein instabiler Thorax, welcher mit einer Sterblichkeit von 33 % assoziiert ist [3]. Die Häufigkeit der Rippenfraktur nach CPR ist in der Alterspopulation über 40 Jahre mit 31 % vergleichsweise hoch [4]. Thompson et al. konnten anhand von 236 Patienten *post mortem* nach CPR zeigen, dass 14,8 % einen instabilen Thorax, wie oben definiert, aufwiesen. Davon fielen die meisten Fälle auf das Kollektiv der 60- bis 79-Jährigen [4]. Dies zeigt sich auch in den bekannten Fallbeschreibungen, in denen 4 von 6 Patienten zu diesem Kollektiv gehören (Tab. 1). Oben genannte Vorteile der Osteosynthese bei Rippenfrakturen könnten insbesondere in dieser Altersgruppe eine Behandlungsverbesserung erzielen [5]. Zu beachten sind bei CPR-assoziierten Frakturen die Sternumquerfrakturen, welche mit einer Häufigkeit bis zu 80 % nach CPR auftreten und zu einem instabilen Thorax und einer inversen Atmung beitragen können [6]. Hier kann auch eine selektive Rippenverplattung das frakturierte Sternum in seiner anatomischen Position halten und den mitfrakturierten kontralateralen Hemithorax mechanisch unterstützen. Die radiologische Bildgebung sollte immer in Kombination mit dem klinischen Bild des Patienten gesehen werden. So zeigt sich im vorgestellten Fall eine undislozierte Serienfraktur des rechten Hemithorax, die in Kombination mit der Sternumfraktur zu einer klinischen Instabilität des gesamten anterioren Thorax beiträgt (Zusatzmaterial online: Video 1). Reanimationsverletzungen sind als punktueller direkter Traumamechanismus zu werten und resultieren in Kombinationen aus Sternumfraktur und Rippenserienfrakturen. Bei symptomatischen Frakturen, instabilem Thorax und sternovertebralen Verletzungen ist die Indikation zur zusätzlichen Verplattung großzügig zu stellen [7]. Plattenosteosynthesen mit „low profile design“ haben sich als patientenfreundlich erwiesen [8]. In den hier dargestellten Fällen zeigte sich dieses Frakturmuster in 3 von 6 Fällen. In der Literatur beschriebene CPR-assoziierte Verletzungen thorakaler Organe, wie z. B. Verletzungen der Trachea oder Lungenlazerationen, sind bei der Indikationsstellung zur Osteosynthese der Rippen zu beachten, und ggf. ist zeitgleich eine videoassistierte Thorakoskopie durchzuführen. Die Übersicht (Tab. 1) zeigt, dass ein höheres Alter mit einer erhöhten Mortalität einhergeht. Eine interdisziplinäre Evaluation von Patienten mit Rippenserienfraktur nach CPR erfolgt nur selten und könnte Morbidität und Mortalität reduzieren. Dies muss mit den Risiken des reanimierten Patienten abgewogen werden. Häufig wird hier eine Koronarintervention mit Stent-Implantation und einer doppelten Thrombozytenaggregationshemmung durchgeführt. Daher finden mögliche Operationen unter erhöhtem Blutungsrisiko oder dem Risiko der Stent-Thrombose statt [9].

**Table 1:** 

Referenz	Alter, Geschlecht	Nebendiagnosen	Anzahl der betroffenen Rippen	Indikation zur Osteosynthese	Osteosynthese nach Tagen ab CPR	Anzahl an CPR (*n*)	Beatmung nach Osteosynthese (Tage)/Beatmungsform	Verlauf	Komplikationen
Olga Ananiadou et al. [10]	59, m	Myokardinfarkt, Herzinsuffizienz (EF 15 %), Hemiplegie, Polyneuropathie	Sternumfraktur Rippen 4 + 5 links	Instabiler Thorax und inverse Atmung	7	2	3/Tracheostoma	Spontanatmung nach 3 Tagen postoperativ	Nicht bekannt
								Nach 3 Wochen Tracheostoma ex	
								Nach 6 Monaten gute Lungenfunktion	
Andrew Drahos et al. [11]	59, m	Nicht bekannt	Rippen 5. + 6. rechts Rippen 3.–6. links	Instabiler Thorax	17	1	16/Tracheostoma	Rehabilitationsbehandlung	Nicht bekannt
								Einen Monat nach Krankenhausentlassung Besserung der Lungenfunktion	
Pouwels et al. [12] Fall 1	79, m	Nicht bekannt	Rippen 3–6 rechts Rippen 4–7 links	Inverse Atmung	14	1	14	Tod nach unklarem Infekt	Spannungspneumothorax
Pouwels et al. [12] Fall 2	63, m	LAE	Sternumfraktur Rippen 2–7 rechts Rippen 3–7 links	Instabiler Thorax	4	2	1/Intubation	Eine Woche Krankenhausaufenthalt post extubationem	Nicht bekannt
								Rehabilitation	
								Gute Lungenfunktion	
Taketani et al. [13]	69, m	Kolon-CA Niereninsuffizienz	Sternumfraktur Rippen 2–5, 7, 8 rechts Rippen 2–7 links	Instabiler Thorax, inverse Atmung, Schmerzen, erhöhte Atemarbeit	22	Unbekannt	4/Tracheostoma	Nicht bekannt	Nicht bekannt
Präsentierter Fall	69, m	Art. Hypertonus Dyslipidämie Z. n. Nikotinabusus (30 PY) Adipositas Eingefäß-KHK	Rippen 2–5 rechts, Stückfraktur 4. Rippe links Dislozierte Frakturen der 3, 5–7 Rippen links	Inverse Atmung	10	3	4/Intubation	Intensivaufenthalt für 5 Tage nach Extubation	Mechanischer Ileus Peritonitis
								Anschließend Rehabilitation	

## Fazit für die Praxis

- Rippenfrakturen sind häufige Begleittraumata nach kardiopulmonaler Reanimation v. a. bei älteren Patienten.
- Das Auftreten einer inversen Atmung bei instabilem Thorax oder starke atemabhängige Schmerzen kann/können eine osteosynthetische Versorgung indizieren.
- Durch Rippenosteosynthese können sekundäre Folgen einer verlängerten mechanischen Beatmung reduziert werden.
- Ein interdisziplinärer Austausch ist nötig, um geeignete Patienten dieser Therapie zuzuführen.

## Supplementary Information




